# A Half-Sandwich Os(II) Glucoconjugated NHC Complex as a Modulator of Amyloid Aggregation

**DOI:** 10.1021/acs.inorgchem.4c04823

**Published:** 2025-02-13

**Authors:** Daniele Florio, Alfonso Annunziata, Valeria Panzetta, Paolo A. Netti, Francesco Ruffo, Daniela Marasco

**Affiliations:** a IRCCS SYNLAB SDN, Via G., Ferraris 144, Naples 80146, Italy; b Department of Chemical Sciences, University of Naples Federico II, Naples 80126, Italy; c Department of Chemical, Materials, and Industrial Production Engineering (DICMaPI), University of Naples Federico II, Naples 80125, Italy; d Interdisciplinary Research Centre on Biomaterials (CRIB), University of Naples Federico II, Istituto Italiano di Tecnologia, Naples 80125, Italy; e Department of Pharmacy, University of Naples Federico II, Naples 80131, Italy

## Abstract

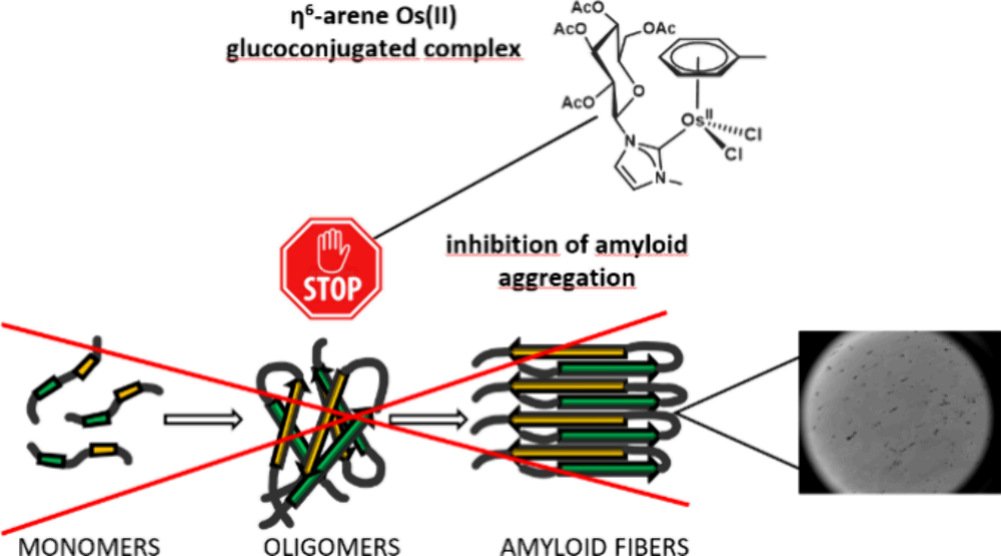

Herein, the effects of a novel half-sandwich Os(II) complex on the aggregation of an amyloid model system, derived from the C-terminal domain of the nucleophosmin 1 protein (NPM1_264–277_), were investigated. The thioflavin T (ThT) binding assay revealed that the complex [(η^6^-toluene)Os(NHCglu)Cl_2_] (where NHCglu is the N-heterocyclic carbene ligand 1-methyl-3-{2,3,4,6-tetra-*O*-acetyl-1-glucosyl}imidazol-2-ylidene), hence named **Os-Tolu**, was able to repress amyloid aggregation in a dose-dependent way. Conformational studies through circular dichroism (CD) and Fourier transform infrared (FTIR) spectroscopies clearly indicated that this inhibitory effect occurred through the stabilization of α-helical structures of monomeric NPM1_264–277_, thus hampering self-recognition. Electrospray ionization mass spectrometry (ESI-MS) studies evidenced, through the formation of coordination adducts, direct interactions of the amyloid peptide with the Os-glucoconjugate complex that, in turn, promote chemical modifications of the sequence further disfavoring the self-assembly process. Noticeably, the presence of **Os-Tolu** completely repressed the formation of amyloid fibers in scanning electron microscopy (SEM) analysis and induced a slight rescue of cell viability, in contrast to its reduction caused by the amyloid model in human SH-SY5Y neuroblastoma cells. These data strongly support the hypothesis of expanding the use of osmium-based agents to neurodegenerative diseases, positioning them as potential neurodrugs.

## Abstract 2

The η^6^-arene Os(II) NHC complex has demonstrated effectiveness in inhibiting the amyloid aggregation of the NPM1_264−277_ peptide, assumed as an amyloid model. This complex acts as an inhibitor of aggregation in the initial steps of the amyloid process, effectively suppressing fiber formation and reducing amyloid toxicity by recovering cell viability. These findings underscore its potential application as a novel neurodrug.

## Introduction

In the last decades, the use of transition-metal-based drugs has been extensively explored in medicinal chemistry,^1^ and currently, several efforts are being performed to design metallodrugs with high efficacy, improved pharmacological properties, and broad-spectrum activity.^2−4^ Metal-based drugs are valid tools to fight amyloid diseases,^5−8^ a family of pathologies involving the formation of toxic oligomers and fibrillar plaques in the brain through the aggregation of amyloid protein/peptides.^9^ We^10,11^ and others^12−15^ studied Pt(II), Ru(II),^16−18^ and Ru(III)^19−23^ complexes as amyloid inhibitors through a “drug repurposing” approach concerning the employment of known anticancer agents. Glucoconjugation is a promising strategy to improve the bioactivity of organometallic complexes since the sugar moiety offers significant advantages in terms of aqueous solubility and overall biocompatibility.^24−26^ Accordingly, we recently reported that glucoconjugated half-sandwich Ru(II) complexes, of general formula [(η^6^-arene)Ru(NHC_glu_)Cl_2_], are valid inhibitors of amyloid aggregation.^27,28^ Herein, the NHCglu ligand was chosen again but with another metal ion, Os(II), to speculate different effects driven by the chemistry of the metal in the complex.

Indeed, despite the structural similarity to ruthenium compounds, osmium complexes usually display greater kinetic inertness, easier accessibility to higher oxidation states, and marked π-back-donation properties in low oxidation states.^29^ In recent years, they received ever-increasing attention in bioinorganic chemistry.^30−34^

Half-sandwich Os(II) arene complexes have attracted much interest for their chemical and biological properties.^35−39^ Noteworthily, the Sadler group observed that, in aqueous systems, the hydrolysis of the Os–Cl bond in Os(II) complexes with formula [(η^6^-biphenyl)Os(en)Cl] (en = ethylenediamine) was slower than that of the Ru(II) analogue, and the resulting aqua-adduct was more acid.^40^ A comprehensive biological investigation on a derivative of formula [Os(η^6^-*p*-cymene)(4-(2-pyridylazo)-*N*,*N*-dimethylaniline)I]PF_6_ showed high cytotoxicity toward many cancer cell lines and promising activity in vivo, probably with a mechanism of action (MOA) different from cisplatin.^41^ Later on, Os-based Noyori-type^42^ catalysts were found to be active for asymmetric transfer hydrogenation inside cells.^43,44^ Recently, the Pizarro group demonstrated that the replacement of the chelating N,N- or N,O- ligands with C,N-cyclometalated ligands (C,N = phenylpyridine) in half-sandwich organoosmium(II) complexes produced highly basic aqua-adducts with a cytotoxicity correlated to thioredoxin reductase inhibition.^45^ The same group also reported that the introduction of hemilabile tether alkoxy ligands in half-sandwich Os(II) complexes is a valid strategy to tune the biological reactivity: some of the complexes were able to catalyze the conversion of pyruvate to lactate.^46^ Such a rich scenario can inspire the development of half-sandwich osmium(II) organometallic complexes in inorganic medicinal chemistry.

**Figure 1:**
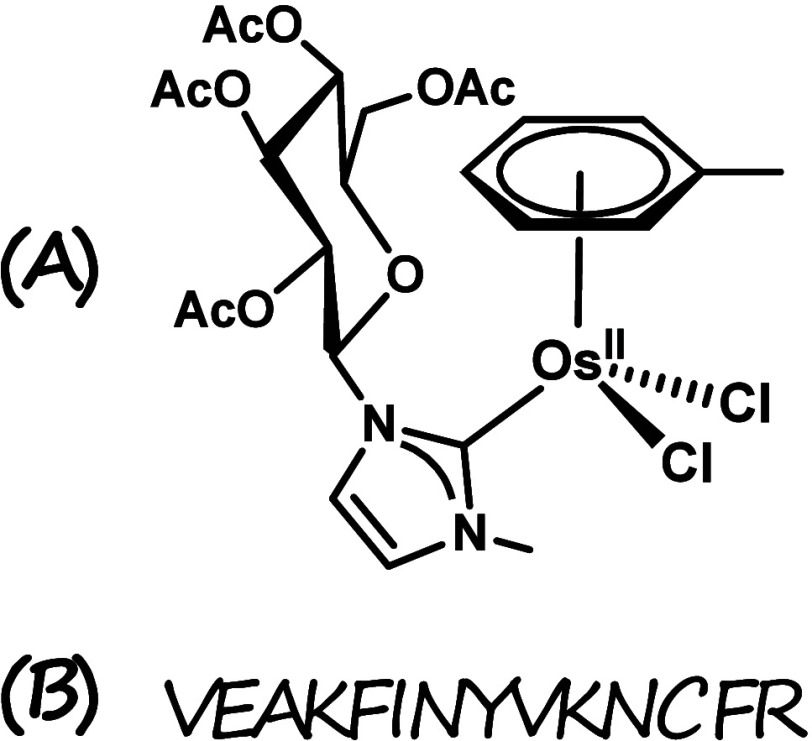
(A) Structure of the Os(II) NHC complex **Os-Tolu**. (B) Primary structure of NPM1_264–277_.

Based on these and on our previous studies,^27,28^ we investigated the activity of a novel half-sandwich Os(II) complex of formula [(η^6^-toluene)Os(NHC_glu_)Cl_2_] named **Os-Tolu** (Figure 1).

Amyloid-forming proteins and peptides are essential tools in drug discovery for neurodegenerative diseases, enabling the screening and identification of modulators that target amyloid aggregation. Often, peptides are used as structural models of amyloid even if they are not directly linked to neurodegenerative conditions.^47−49^ In this context, a notable example is represented by a fragment of nucleophosmin 1 (NPM1): although not a neurodegenerative protein, it contains a well-characterized amyloidogenic region in the second helix of its C-terminal domain, NPM1_264–277_,^50^ which has been already used as an amyloid model.^16,28,51^

In this study, the **Os-Tolu** complex was synthesized and fully characterized by spectroscopic techniques, and its activity as an inhibitor of amyloid aggregation of NPM1_264–277_ was evaluated by several spectroscopic, biochemical, and biophysical techniques, aiming for the development of osmium compounds as novel antiamyloid therapeutics.

## Results and Discussion

### Synthesis and Characterization of **Os-Tolu**

**Scheme 1:**
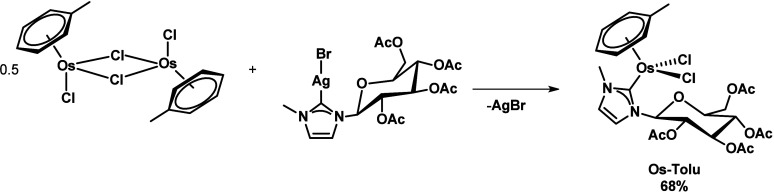
Synthetic Route to Access **Os-Tolu**

In general, glycoconjugated metallodrugs^52−56^ display favorable properties including an enhancement of intracellular accumulation through the Warburg Effect.^57^ NHC_glu_ proved to be a highly versatile ligand enabling the synthesis of glucoconjugated organometallic derivatives of Pt,^58−60^ Au,^61,62^ and Ru.^63^ Following a similar synthetic strategy, **Os-Tolu** was obtained by a transmetalation reaction between the dimeric precursor [Os(η^6^-tolueneCl_2_]_2_ and 2 equiv of the glucoconjugate silver carbene (Ag-NHC_glu_)^58^ in acetone. After removing the precipitated AgBr, the compound was crystallized by the addition of diethyl ether (Scheme 1).

**Figure 2:**
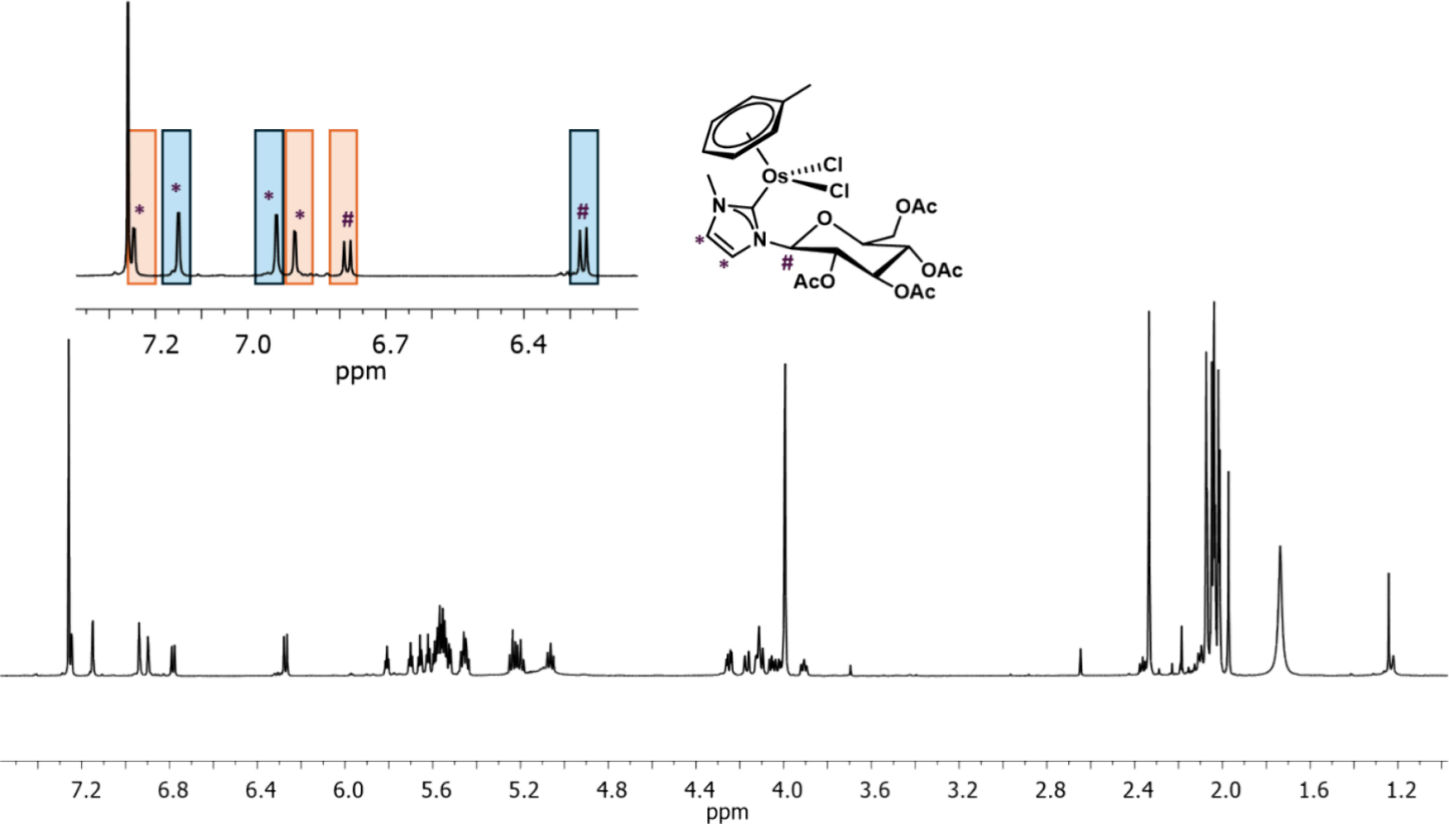
^1^H NMR spectrum of **Os-Tolu** in CDCl_3_ at 263 K. In the inset, the highlighted signals are attributed to the NHC imidazole (*) and the anomeric glucosyl (**#**) protons.

**Os-Tolu** was soluble in a wide range of organic solvents including acetone, DCM, chloroform, DMSO, methanol, and water (up to ca. 50 mM). ^1^H NMR spectra recorded in CDCl_3_ and CD_3_OD display a broadening of signals at room temperature (Figures S1 and S2), which was resolved by recording the spectra at 263 K (Figure 2).

At this temperature, the spectra show the presence of two distinct sets of signals attributable to two different species in a ratio 1:1.25. These were identified as two rotamers probably in equilibrium through the rotation around the Os–C(NHC) bond and diastereomeric due to the chirality of the sugar residue. A similar behavior was observed for other chiral half-sandwich NHC complexes and in our analogue glucosylated Ru(II)-NHC compounds.^63^ However, the two species display similar spectral profiles, with the aromatic protons of the π-bound toluene upfield shifted at 5–6 ppm with respect to free toluene, the sugar protons resonating at the expected frequencies with the typical coupling constant (^3^*J)* patterns of a ^4^C_1_ glucosyl ring in β-configuration.^64^ The complex was further characterized by recording ^1^H–^1^H COSY (Figure S3) and ^13^C (Figure S4) NMR spectra at 263 K in CDCl_3_. The carbene carbon atoms resonated at 162 and 164 ppm, in line with the chemical shift observed for other η^6^-arene Os(II) complexes.^65,66^ The hydrolytic behavior of the complex in aqueous systems was assessed by UV–vis spectroscopy and ESI-MS. The UV–vis spectra of **Os-Tolu** in water over 5 h (Figure S5A) revealed a substantial lack of variation of bands, confirming the stability of the complex in the investigated interval time. ESI-MS (see Materials and Methods for details) suggested the hydrolysis of the Os-Cl coordination to form reactive aqua-adducts.^63^

### Fluorescence Studies: Thioflavin T (ThT) and Autofluorescence

**Figure 3:**
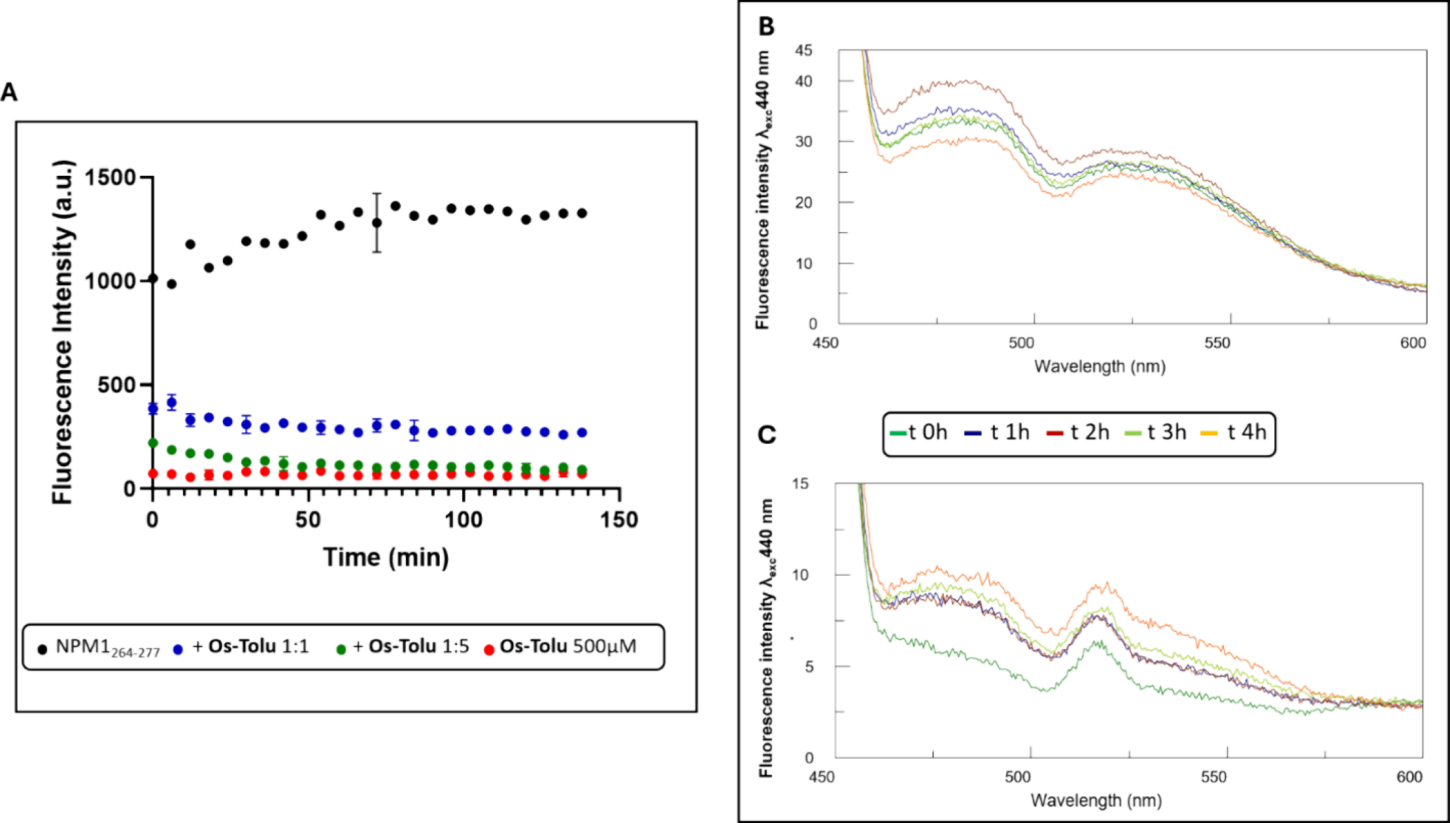
Left panel: (A) ThT fluorescence assay of NPM1_264–277_ alone (black) and NPM1_264–277_ in the presence of **Os-Tolu** 1:1 (blue) and 1:5 (green). **Os-Tolu** alone is reported in red. Right panel: overlay of florescence emission spectra: λ_exc_= 440 nm of (B) NPM1_264–277_ alone and (C) NPM1_264–277_ in the presence of **Os-Tolu** 1:5.

To analyze the effects of the **Os-Tolu** complex on the amyloid aggregation of NPM1_264–277_, the thioflavin T (ThT) assay was employed. The overlays of fluorescence emission profiles in the presence or absence of the **Os-Tolu** complex are reported in Figure 3A.

The NMP1_264–277_ peptide at *t* = 0 displayed a no zero value, which indicated an already aggregated state despite HFIP treatment (see Materials and Methods).

The presence of the complex caused a neat decrease of the ThT signal at early stages of aggregation in a dose-dependent way: the equimolar condition (1:1 ratio) caused an inhibition of ∼60%, while the 1:5 ratio resulted in a major suppressive effect with a reduction in signal of ∼80%. The autofluorescence assay for amyloid systems is emerging as a crucial method for assessing the modulatory impact of external agents on amyloid aggregation.^67^ The effect of the **Os-Tolu** complex on the autofluorescence of NPM1_264–277_ was assessed by recording fluorescence emission spectra over time with excitation at 440 nm (Figure 3B). In these conditions, NPM1_264–277_ alone displayed two emission maxima at 478 and 525 nm, as previously reported.^51^ Afterward, a slight increase in signal after 2 h of aggregation was followed by a decrease in intensity up to 4 h. In the presence of the **Os-Tolu** complex, a drastic reduction of blue fluorescence was observed. As a control experiment, the Os complex alone showed a signal not comparable to that exhibited by NPM1 _264–277_ alone and quite similar to that in the presence of the amyloid peptide (Figure S5B).

### Conformational Studies: CD and FTIR Spectroscopy

**Figure 4:**
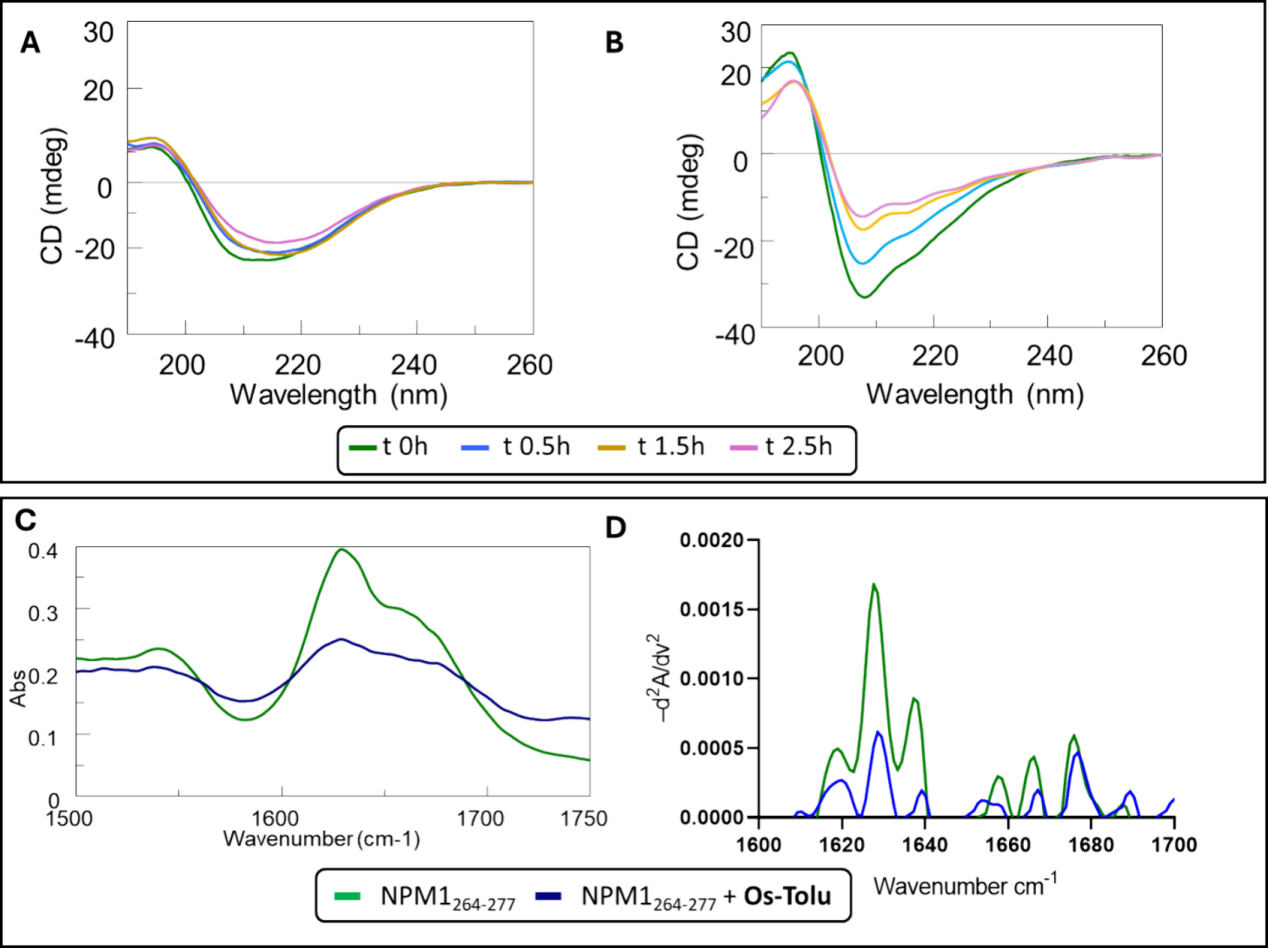
Upper panel: overlay of CD spectra over time of (A) NPM1_264–277_ and (B) NPM1_264–277_ + **Os-Tolu** 1:1 molar ratio. Lower panel: FTIR spectra of (C) NPM1_264–277_ alone and in the presence of **Os-Tolu** 1:1 molar ratio and (D) deconvoluted and second-derivative FTIR spectra.

To assess whether the presence of the **Os-Tolu** complex could influence the conformational features of the NPM1_264–277_ peptide, CD spectra of the peptide alone and in the presence of the Os complex were recorded at different times. The overlays of the spectra are reported in the upper panel of Figure 4.

CD profiles already exhibited distinct features between the peptide alone (Figure 4A) and in the presence of the **Os-Tolu** complex (Figure 4B) at *t* = 0. As already reported,^50^ NPM1 _264–277_ exhibited CD spectra with a broad band centered at ∼218 nm, suggesting mixed α + β conformations, where the Cotton effect slightly decreased during the time for aggregation.^50^ The presence of the **Os-Tolu** complex determined the onset of a maximum at 190 nm and two minima at 208 and 220 nm, respectively, that are typical of α-helix conformation, which is the native conformation of this sequence in the protein architecture.^68^ These considerations were confirmed by the deconvolution data reported in Table 1 showing a clear stabilization of the α-helix structure at early stages in the presence of the **Os-Tolu** complex. As control CD signal of the complex alone, reported in Figure S6A, indicated a negligible CD signal.

**Table 1:** Deconvolution of CD Spectra of NPM1_264-277_ Alone and in the Presence of **Os-Tolu** at a 1:1 Molar Ratio Reported in Figure 4A,B at the Indicated Times

	**time (h)**	α-helix	β-sheet	**turn**	**others**
NPM1_264–277_	0	34.7	20.7	8.0	36.6
	0.5	29.3	25	9.4	36.3
	1.5	21.5	24.3	10.8	43.4
	2.5	28.3	26.5	9.3	35.9
NPM1_264–277_ +**Os-Tolu**	0	41.9	10.7	4.6	42.8
	0.5	36.8	7.4	7.6	48.1
	1.5	28.5	9.9	10.7	50.8
	2.5	19.8	11.5	11.8	56.9

FTIR spectroscopy confirmed the α-helical stabilization effect of the **Os-Tolu** complex (Figure 4, lower panel). The IR spectra of NPM1_264–277_ alone and in the presence of the **Os-Tolu** complex were analyzed after 2 h of stirring. NPM1 _264–277_ alone contained β-sheets with the presence of a dominant peak at ∼1630 cm^–1^ and a second shoulder at 1680 cm^–1^ (Figure 4C), indicating an antiparallel orientation of the β-strands.^69^ The analysis of second derivative spectra (Figure 4D) allowed the deepening of secondary structures,^70^ and deconvoluted data (Table 2) indicated that NPM1_264–277_ alone contained β-sheet structures, while the presence of the **Os-Tolu** complex promoted a conformational stabilization of the α-helix conformation. This is evidenced by the characteristic α-helix band at 1656 cm^–1^ corresponding to hydrogen-bonded C=O stretches in the α-helix conformation.

**Table 2:** Deconvoluted Amide I Band Frequencies and Assignments to the Secondary Structure for NPM1_264-277_ Alone and in the Presence of **Os-Tolu** at a 1:1 Molar Ratio

	**mean frequencies**	**assignment**
NPM1_264–277_	1642 ± 1.0	β-sheet
NPM1_264–277_ + **Os-Tolu** complex	1656 ± 2.0	α-helix

### ESI-MS Analysis of the Adducts between **Os-Tolu** and NPM1_264–277_

**Figure 5:**
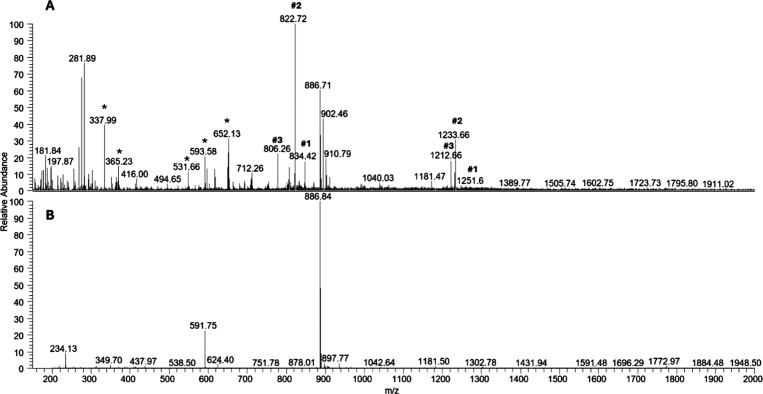
ESI-MS spectra of (A) NPM1_264–277_ + **Os-Tolu** 1:1 molar ratio and of (B) NPM1_264–277_ alone reported as reference. Asterisks (*) highlight the species present in the complex alone.

We analyzed the formation of potential adducts between the **Os-Tolu** complex and NPM1_264–277_ peptide through electrospray ionization mass spectrometry (ESI-MS): mass spectra were registered after 2 h of stirring and are reported in Figure 5. The complex alone was analyzed as the control, and the related spectrum is reported in Figure S6B.

In the presence of **Os-Tolu**, many additional peaks were evident due to the formation of adducts between the complex and the peptide with a 1:1 stoichiometry (Figure 5 and Table 3). Specifically, peak #1 (*m*/*z* = 1251.6 and 834.32 a.m.u.) was due to the formation of an adduct peptide/metal complex with the loss of one chloride ligand, peak #2 (*m*/*z =* 1233.66 and 822.72 a.m.u.) was from the same adduct with the loss of the second chloride ligand, and peak #3 (*m*/*z* = 1212.66 and 806.22 a.m.u.) was from the same product with the additional loss of one acetyl group of the sugar moiety. In the identification of different species, several peaks are consistent with dual Cys ^275^ oxidation as already reported,^51^ with the occurrence of signals at *m*/*z* 902.46 and 910.79 a.m.u. (Table 3) corresponding to the dioxidated form of Cys as sulfinic acid^71^ (2ox NPM1_264–277_) and to the trioxidized form of Cys as sulfonic acid^72^ (3ox NPM1_264–277_).

**Table 3:** Table of the Main Observed MS Peaks Formed by NPM1_264–277_ in the Absence and in the Presence of the **Os-Tolu** Complex after 2 h of Stirring

**description**	*m*/*z***(charge)**	**theoretical***m*/*z*
NPM1_264–277_	1772.42	1772.08
NPM1_264–277_	886.84 (+2)	887
NPM1_264–277_	591.75 (+3)	591.66
2ox NPM1_264–277_	902.46 (+2)	902.48
3ox NPM1_264–277_	910.79 (+2)	910.48
NPM1_264–277_ + **Os-Tolu**-1Cl (#1)	1251.6 (+2)	1251.5
NPM1_264–277_ + **Os-Tolu**-1Cl (#1)	834.32 (+3)	834.66
NPM1_264–277_ + **Os-Tolu**-2Cl (#2)	1233.66 (+2)	1234.00
NPM1_264–277_ + **Os-Tolu**-2Cl (#2)	822.72 (+3)	823.00
NPM1_264–277_ + **Os-Tolu**-2Cl- 1Ac (#3)	1212.66 (+2)	1212.04
NPM1_264–277_ + **Os-Tolu**-2Cl- 1Ac (#3)	806.22 (+3)	808.69

To corroborate our assignment, we compared the experimental and simulated isotopic pattern of several *m*/*z* values (Figure S7 and Table S1), with good agreement.

### Effects of **Os-Tolu** on the Morphologies of NPM1_264–277_ Derived Fibers

**Figure 6:**
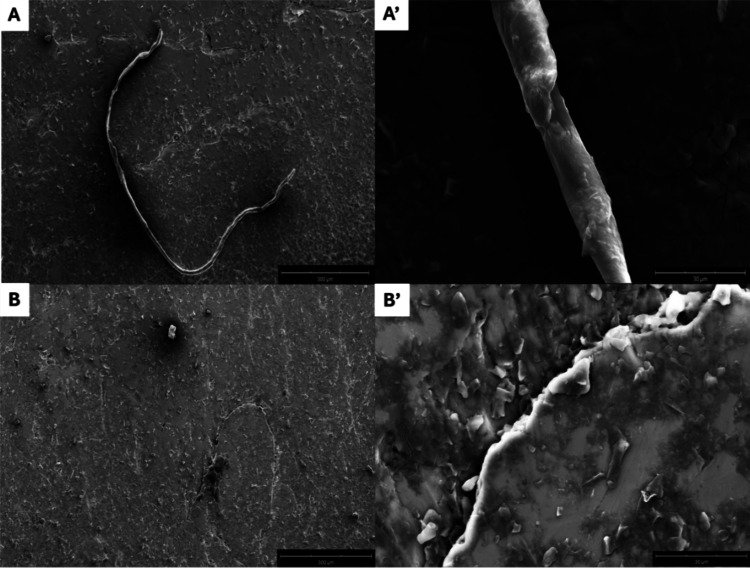
SEM micrographs of NPM1_264–277_ (100 μM) alone (A, A′) and in the presence of **Os-Tolu** (B, B′) at 1:1 peptide/metal complex molar ratio. Overviews of the surface of the samples at 300 μm (A, B) and 30 μm (A′, B′).

To get insights into potential variations of the morphology of the aggregates derived from the NPM1_264–277_ peptide in the presence and in the absence of **Os-Tolu**, scanning electron microscopy (SEM) analysis was used. The samples were analyzed after 4 h of aggregation, and the resulting micrographs are shown in Figure 6. As can be noticed, while the peptide alone produced a well-defined fiber,^73^ the presence of the **Os-Tolu** complex significantly affects the formation of the amyloid fibers, leading to their total suppression (Figure 6 B,B′). As a control, under the same experimental conditions, the SEM of the complex alone, reported in Figure S6C, did not highlight the presence of any aggregate.

### Cellular Effects of **Os-Tolu** on the Amyloid Cytotoxicity of NPM1_264–277_

**Figure 7:**
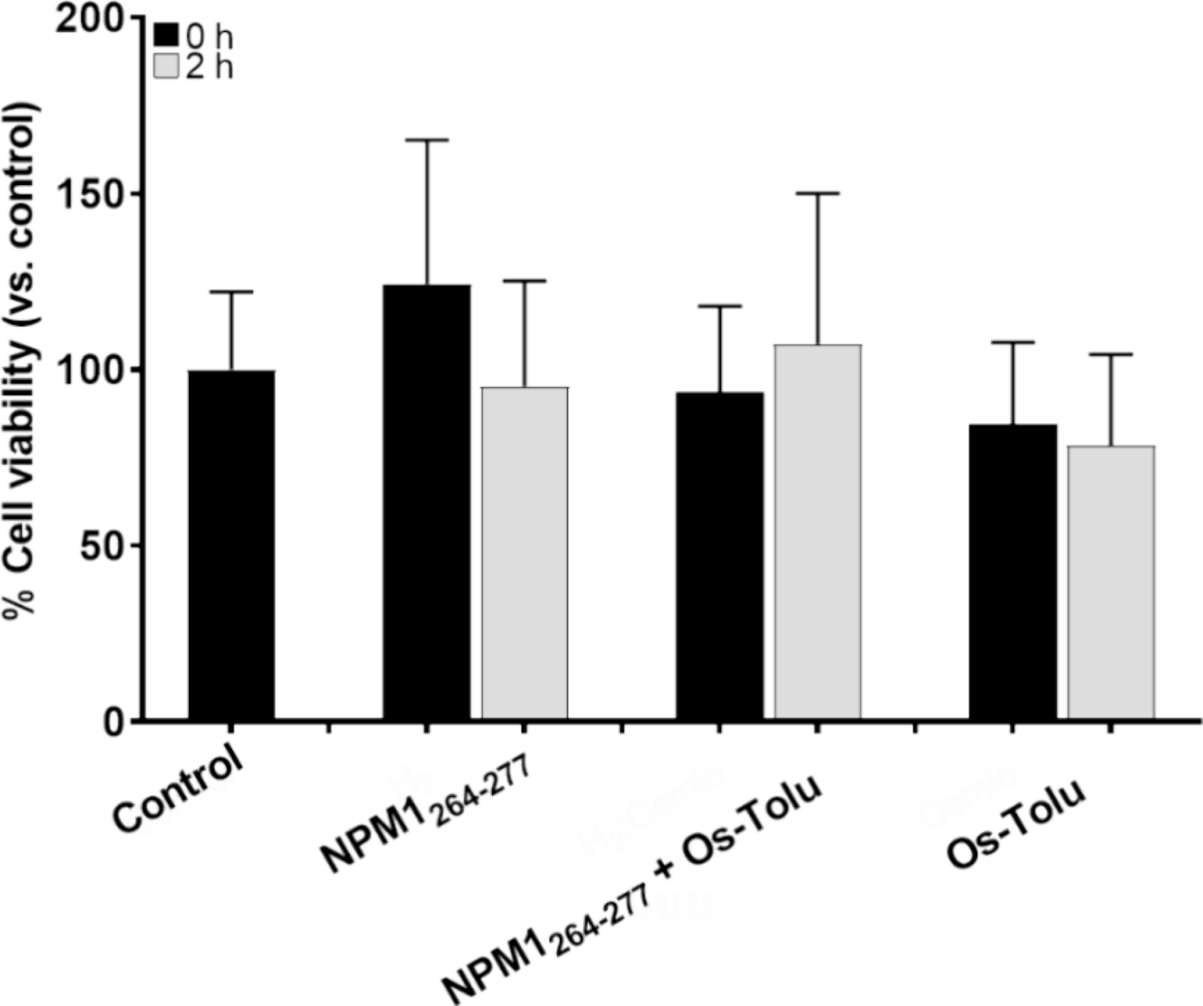
Effects of **Os-Tolu** on the cytotoxicity of the NPM1 _264–277_ peptide in SH-SY5Y neuroblastoma cells: the MTT assay of NPM1 _264–277_ peptide in the absence and in the presence of **Os-Tolu**, incubated under stirring, at *t* = 0 and 2 h. Control refers to untreated cells as 100% of viable cells. Data are reported as mean ± standard deviation. Statistical comparisons were performed with a Student’s unpaired *t*-test.

To determine whether **Os-Tolu** can protect against amyloid-induced cytotoxicity, we conducted an MTT assay on SH-SY5Y cells at two time points: 0 and 2 h of aggregation. SH-SY5Y cells were treated with the peptide NPM1_264–277_ alone and with the peptide NPM1_264–277_ in the presence of the **Os-Tolu** complex in a 1:1 molar ratio. The histogram of cell viability is reported in Figure 7. As already observed, NPM1_264–277_ reduced cell viability by approximately 20% after 2 h of aggregation; in contrast, the presence of the **Os-Tolu** complex showed a trend toward a recovery of cell viability at the same time. The **Os-Tolu** complex alone displayed a level of cytotoxicity comparable to that of the peptide NPM1_264–277_ after 2 h of aggregation, although these variations were not significant.

## Conclusions

A substantial number of osmium complexes, featuring the metal in various oxidation states (II, III, IV, VI),^30,32,34,35,74^ displayed notable antiproliferative activity against cancer cell lines. Organoosmium(II) arene compounds show a wide range of biological activities in which the charge and the steric hindrance of ligands exert a primary role in substitution-hydrolysis processes, and osmium reactivity can be finely tuned by selecting appropriate chelating ligands.^75,46,76^ In the present study, an Os(II) complex with a η^6^-toluene, a glucoconjugated NHC, and two chlorides as ligands, named **Os-Tolu** (Figure 1), was investigated for its ability to interfere with the aggregation, fibrillation, and cytotoxicity of the amyloid model corresponding to the fragment NPM1_264–277._

By the ThT assay, we evaluated the ability of **Os-Tolu** to almost abolish the formation of amyloid aggregates even at a 1:1 molar ratio. This inhibition translated into a complete suppression of amyloid fibers in the presence of the metal complex, as detected by SEM analysis, and provided a tendency to rescue cellular viability in SH-SY5Y cells. ESI-MS investigations suggested the formation of coordination adducts with the loss of chlorides around the metal ion. Additionally, it should also be underlined that the redox activity of the complex can exert a primary role in its MOA: our data clearly indicate that, in the case of the amyloid sequence containing cysteines, as NPM1 _264–277_, this complex can promote, stoichiometrically or catalytically, the progressive oxidation of thiols as already reported for similar complexes.^37,77^ The presence of sulfoxide and sulphone species can create a more hydrophilic environment^23,78^ that limits the self-recognition mechanism among monomers that is the basis of the aggregation process. This result can explain the stabilization of an α-helical conformation of the NPM1_264–277_ monomer. The α-helix is the native conformation of the sequence 264–277 in NMP1,^68^ and this is one of the few examples of a metallodrug that inhibits the amyloid aggregation of NPM1_264–277_ by fixing its helical conformation, as assessed through both CD and FTIR spectroscopies. This evidence also confirms our speculation concerning the fact that the α-helix is “a protective” conformation adopted by the NPM1 C-terminal domain to avoid the aggregation^50^ of the wild-type protein that instead occurs in AML mutated versions of this protein.^79−82^ Indeed, recently, we performed an analogue study employing Ru-based complexes quite similar to the Os compound analyzed in the present investigation:^28^ data indicated similar ThT signal reductions and the formation of a coordination adduct between NPM1 _264–277_ and the metal complex with the release of the same ligands. In addition, in the case of the Os(II) complex, a more pronounced helical content with respect to that containing Ru(II) ion was observed.

The neat positive charge of the aqua-species derived from Os-Cl hydrolysis in **Os-Tolu** favored its interaction with a peptide sequence with a high pI value, ∼10, such as NPM1_264–277_.^50^ The present investigation is, to the best of our knowledge, only the second example of the employment of the Os(II) complex present in the literature.^83^ Indeed, in a concomitant study, a piano-stool osmium complex revealed the ability to inhibit Aβ aggregation even with different MOAs. In this case, the complex presents a different structure, i.e., [(η^6^-*p*-cymene)Os**L**Cl]PF_6_, where L is a bidentate chelating quinoline benzimidazole-based ligand. ESI-MS did not provide indications on the formation of any coordination adduct between the Os complex and the peptide upon ligand substitutions around the metal ion. In that case, the Os complex did not completely abolish the formation of amyloid fibers, and the required presence of DMSO (at 5% (v/v)), likely due to the lack of hydrophilic ligands as glucosyl,^84^ could limit its translation as a neurodrug, confirming that the design of the coordination environment of metallodrugs has a crucial impact on the chemical properties and the biological effects in medicinal inorganic chemistry.

In preliminary investigations, **Os-Tolu** was revealed to be able to suppress the aggregation of Aβ_1–42_, but further ongoing studies are required to deepen differences in the MOAs of **Os-Tolu** toward diverse amyloid systems. In conclusion, this study highlights the potentialities of Os-based metallodrugs in the field of amyloid aggregation and can pave the way to their different employment in innovative neurodiagnostic and neurotherapeutic strategies.

## Experimental Section

### Materials and Methods

Reagents and solvents were used as received from commercial suppliers. [Os(η^6^-tolueneCl_2_]_2_ and Ag-NHC_glu_^58^ were prepared according to literature procedures. NMR spectra were acquired on a Bruker Avance NEO 600 MHz NMR spectrometer equipped with a cryogenically cooled proton-optimized TCI probe. The solvents for NMR studies were CDCl_3_ (CHCl3, δ 7.26, and ^13^CDCl3, δ 77.0, as internal standards) and CD_3_OD (CD_2_HOD, δ 3.30, and 13CD_3_OD, δ 49.0, as internal standards). The following abbreviations were used to describe NMR multiplicity: s, singlet; d, doublet; dd, double doublet; triplet; and m, multiplet.

### Synthesis of the **Os-Tolu** Complex

[Os(η^6^-tolueneCl_2_]_2_ (100 mg, 0.14 mmol) and Ag-NHC_glu_ (183 mg, 0.30 mmol) were stirred protected from light in acetone (10 mL) for 18 h. At the end of this time, the precipitated AgBr was filtered off through a pad of Celite, and the clear filtrate was reduced in volume to ca. 2 mL. Addition of diethyl ether resulted in the precipitation of a yellowish solid, which was washed with ether and dried under a vacuum. Yield: 68%.

^1^H NMR (600 MHz, CDCl_3_) δ 7.25 (d, *J* = 2.3 Hz, 1H, H-Im), 7.15 (d, *J* = 2.2 Hz, 1H, H-Im), 6.94 (d, *J* = 2.1 Hz, 1H, H-Im), 6.90 (d, *J* = 2.2 Hz, 1H, H-Im), 6.78 (d, *J* = 9.5 Hz, 1H, H1-glu), 6.27 (d, *J* = 9.9 Hz, 1H, H1-glu), 5.81 (t, *J* = 5.2 Hz, 1H, H-glu), 5.70 (t, *J* = 5.2 Hz, 1H, H-glu), 5.66 (t, *J* = 5.2 Hz, 1H, H-glu), 5.62 (t, *J* = 5.1 Hz, 1H, H-glu), 5.60–5.51 (m, 7H), 5.45 (td, *J* = 9.4, 6.2 Hz, 2H), 5.24 (t, *J* = 9.2 Hz, 1H), 5.20 (t, *J* = 9.7 Hz, 1H), 5.07 (t, *J* = 7.7 Hz, 1H), 4.25 (dd, *J* = 12.7, 4.6 Hz, 1H, H-glu), 4.17 (dd, *J* = 12.4, 2.0 Hz, 1H, H-glu), 4.14–4.09 (m, 2H, H-glu), 4.08–4.01 (m, 1H, H-glu), 4.00 (s, 3H, Me-Im), 3.99 (s, 3H, Me-Im), 3.91 (ddd, *J* = 9.7, 7.4, 2.0 Hz, 1H), 2.33 (s (x2), 6H, Me-Tol), 2.07 (s (x2), 6H, OAc), 2.05 (s, 3H, OAc), 2.04 (s (x2), 6H, OAc), 2.02 (s, 3H, OAc), 2.01 (s, 3H, OAc), 1.97 (s, 3H, OAc). ^13^C NMR (176 MHz, CDCl3) δ 171.20, 170.61, 170.43 (x2), 169.87 (x2), 169.85, 168.64, 164.13, 162.01, 123.91, 123.26, 119.26, 118.66, 99.27, 98.59, 85.58, 85.41, 79.75, 79.60, 78.74, 78.04, 76.48, 76.44, 76.27, 76.00, 74.05, 73.92, 73.89, 73.10, 70.52, 68.64, 68.57, 68.54, 67.85, 67.68, 63.06, 61.37, 39.93, 39.65, 21.25, 21.12(x2), 21.03, 20.85 (x2), 20.83, 20.72, 18.86, 18.74. ESI-MS(+, MeOH), *m*/*z*: 653.21 [M – 2Cl-OAc], 731.22 [M – Cl]^+^

### Peptide Synthesis

The NPM1_264–277_ peptide was synthesized as already reported^50^ and after purification was treated with 1,1,1,3,3,3-hexafluoro-2-propanol (HFIP) and stored at −20 °C until use.

### Fluorescence Assays

The ThT emission assay was carried out in black plates (96 wells) under stirring on the fluorescence reader Envision 2105 (PerkinElmer) at 25 °C using a ThT concentration of 50 μM and 20 mM borate buffer (pH = 8.5). The peptide was assayed at a concentration of 200 μM alone and in the presence of the **Os- Tolu** complex (stock solution, 50 mM in H_2_O) at 1:1 and 1:5 peptide/metal complex molar ratios. The autofluorescence (λ_ex_ = 440 nm) assay was performed on a Jasco FP 8300 spectrofluorometer at a peptide concentration of 200 μM at 25 °C in 50 mM borate buffer alone and in the presence of the complex. Spectra were registered at indicated times, after stirring, with a scanning speed 50 nm/min.

### Circular Dichroism

CD spectra of NPM1_264–277_ were registered in the far-UV region from 190 to 260 nm on a J-810 spectropolarimeter (JASCO Corp., Milan, Italy) at a concentration of 100 μM in 20 mM borate buffer pH 8.5, alone and in the presence of the **Os-Tolu** complex at a 1:1 peptide to metal complex molar ratio. Spectra of NPM1_264–277_ + **Os-Tolu** were subtracted from the spectrum of **Os-Tolu** alone. A 0.1 cm cuvette at 25 °C was used with the following: scan speed = 20 nm/min, bandwidth= 2.0 nm, resolution = 0.2 nm, sensitivity = 50 mdeg, and response = 4 s. Deconvolution was obtained by employing the BESTSEL software (http://bestsel.elte.hu/).^85^

### FTIR Spectroscopy

Fourier transform infrared spectra of NPM1_264–277_ alone (300 μM) and in the presence of the **Os-Tolu** complex at a 1:1 molar ratio were registered in 50 mM borate buffer at pH 8.5 on a Jasco FT/IR 4100 spectrometer (Easton, MD, USA) in an attenuated total reflection (ATR) mode. The measurements were performed with a Ge single crystal at a resolution of 4 cm^–1^ after 1.5 h of stirring and subsequent drying under a vacuum. A total of 100 scans were recorded for each sample at a rate of 2 mm·s^–1^ against a KBr background. After the collection in transmission mode, the spectra were converted to emission. Amide I deconvolutions were automatically returned as emissions with the built-in software (Spectra Manager 2.5). Deconvoluted amide I band frequencies and secondary structure assignments^70^ for the peptide alone and in the presence of the **Os-Tolu** complex were obtained by evaluating the second-derivative spectrum, which was calculated using a seven-point Savitsky–Golay second-derivative function. The **Os-Tolu** complex alone was analyzed as a control.

### ESI-MS Analysis

Solutions of NPM1_264–277_ at a concentration of 50 μM in 15 mM ammonium acetate (pH = 6.8), alone and in a 1:5 molar ratio with the **Os-Tolu** complex, were incubated for 2 h. The mixtures were diluted 10 times and then analyzed using an LTQ XL Ion Trap mass spectrometer equipped with an electrospray ionization (ESI) source (Thermo Fisher Scientific). Peptides and complexes alone were evaluated as controls.

### SEM Analysis

NPM1_264–277_ alone (100 μM) and NPM1_264–277_ in the presence of the **Os-Tolu** complex (1:1 peptide:/metal complex molar ratio) were morphologically analyzed after 4h of aggregation using field-emission SEM (Phenom_XL, Alfatest, Milan, Italy). After this time, ∼50 μL of the solution was drop-cast on an aluminum stub and dried under a vacuum to prepare the samples. For 75 s, a thin layer of gold was sputtered at a current of 25 mA. Following the introduction of the sputter-coated samples into the specimen chamber, micrographs were taken using a secondary electron detector (SED) at an accelerating voltage of 10 kV. The **Os-Tolu** complex alone (100 μM) was analyzed as a control.

### Cells

SH-SY5Y cells were grown at 37 °C in a humidified atmosphere of 5% CO_2_ in Eagle’s minimum essential medium (EMEM, Sigma-Aldrich MCF7) and Dulbecco’s modified Eagle’s medium and Ham’s F12 (DMEM/F-12, SH-SY5Y) containing 10% fetal bovine serum (FBS), 100 μg mL^–1^l-glutamine, and 100 U mL^–1^ penicillin/streptomycin. SH-SY5Y cells were seeded in duplicates in 96-well plates at a density of 25,000 cells/well and allowed to adhere overnight.

### MTT Assay

NPM1_264–277_ (stock solution 500 μM in 50 mM phosphate buffer at pH 7.4) in the absence and NPM1_264–277_ in the presence of the **Os-Tolu** complex at a 1:1 peptide to metal complex molar ratio (after 0 and 2 h of stirring) were diluted in a cell culture medium at a final concentration of 100 μM and added to the cells for 24 h. Control cells were incubated with phosphate buffer diluted in the cell culture medium at the same final concentration used for the peptide. After the incubation, MTT assays were used according to the manufacturer’s instructions. Briefly, 200 μL of the MTT labeling reagent (final concentration, 0.5 mg/mL; Sigma-Aldrich) was added to each well for 3 h. Then, the supernatant was removed and substituted with 200 μL of isopropanol for 10 min at 37 °C and 5% CO_2_. The optical density of each well sample was determined at 570 nm using a microplate reader. A blank absorbance value of 0.21, obtained from wells without cells but treated with the MTT reagent, was subtracted from all of the absorbance values. Then, the average absorbance value of cells treated with the NPM1_264–277_ peptide was normalized to those of control cells incubated with the buffer, and cell viability was expressed as a percentage of the control.

## Supporting Information Available

^1^H NMR spectrum of **Os-Tolu** in CDCl_3_ at 298 K (Figure S1); ^1^H NMR spectrum of **Os-Tolu** in CD_3_OD at 298 K (Figure S2); ^1^H–^1^H COSY NMR spectrum of **Os-Tolu** in CDCl_3_ at 263 K (Figure S3); ^13^C NMR spectrum of **Os-Tolu** in CDCl_3_ at 263 K (Figure S4); overlay of (A) UV–vis spectra (B) fluorescence emission spectra: λ_exc_= 440 nm of **Os-Tolu** (Figure S5); **Os-Tolu** (A) CD spectrum, (B) ESI-MS spectrum at *t* = 2 h of aggregation and (C) SEM microscopy image at *t* = 4 h of aggregation (Figure S6); comparison of *m*/*z* (*z* = +2) isotopic pattern of experimental (left black panel) and simulated (right red panel) spectra (Figure S7) (PDF)

## ABBREVIATIONS

CD: circular dichroism
DMEM: Dulbecco’s modified Eagle’s medium
EMEM: Eagle’s minimum essential medium
ESI-MS: electrospray ionization mass spectrometry
FBS: fetal bovine serum
FTIR: Fourier transform infrared spectroscopy
HFIP: 1,1,1,3,3,3-hexafluoro-2-propanol
MTT: 3-(4,5-dimethylthiazol-2-yl)-2,5-diphenyltetrazolium bromide
NHC: N-heterocyclic carbene
NPM1: nucleophosmin 1
SEM: scanning electron microscopy
SH-SY5Y: human neuroblastoma cell line
ThT: thioflavin T
