# Supplementary material for: A Half-Sandwich Os(II) Glucoconjugated NHC Complex as a Modulator of Amyloid Aggregation
Source: Inorg Chem. 2025 Feb 13;64(7):3335–45. doi: 10.1021/acs.inorgchem.4c04823 (PMC11863378; doi:10.1021/acs.inorgchem.4c04823)
Supplement: Supplementary file 1 — ic4c04823_si_001.pdf [file ic4c04823_si_001.pdf]

SUPPORTING INFORMATION

A half-sandwich Os(II) glucoconjugated NHC complex as modulator of amyloid aggregation

Daniele Florio[a], *Alfonso Annunziata*[b,†,]*, *Valeria Panzetta*[c,d], *Paolo A. Netti*[c,d], *Francesco Ruffo*[b], *and Daniela Marasco*[e,]*

[a]*IRCCS SYNLAB SDN*, 80146, Naples, Italy.

[b]Department of Chemical Sciences, University of Naples Federico II, 80126, Naples, Italy.

[c]Department of Chemical, Materials, and Industrial Production Engineering (DICMaPI), University of Naples Federico II, 80125 Naples, Italy.

[d]Interdisciplinary Research Centre on Biomaterials (CRIB), University of Naples Federico II, Istituto Italiano di Tecnologia, 80125, Naples, Italy.

[e]Department of Pharmacy, University of Naples Federico II, 80131, Naples, Italy.

***Corresponding Author**

Daniela Marasco - Email: daniela.marasco@unina.it

Alfonso Annunziata - Email: alfonso.annunziata@imdea.org

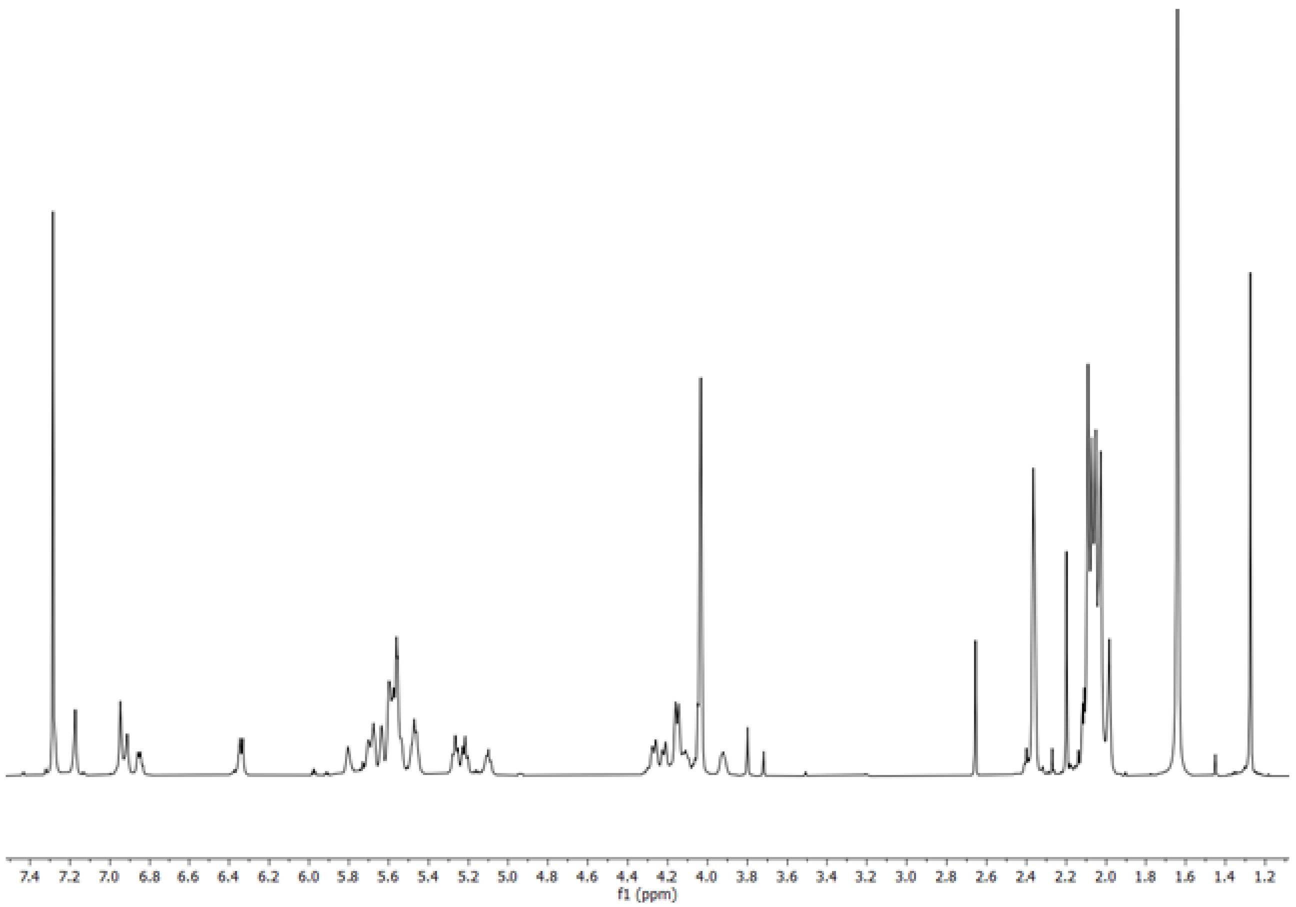

Figure S1. ^{1}H NMR spectrum of **Os-Tolu** in $CDCl_3$ at 298 K.

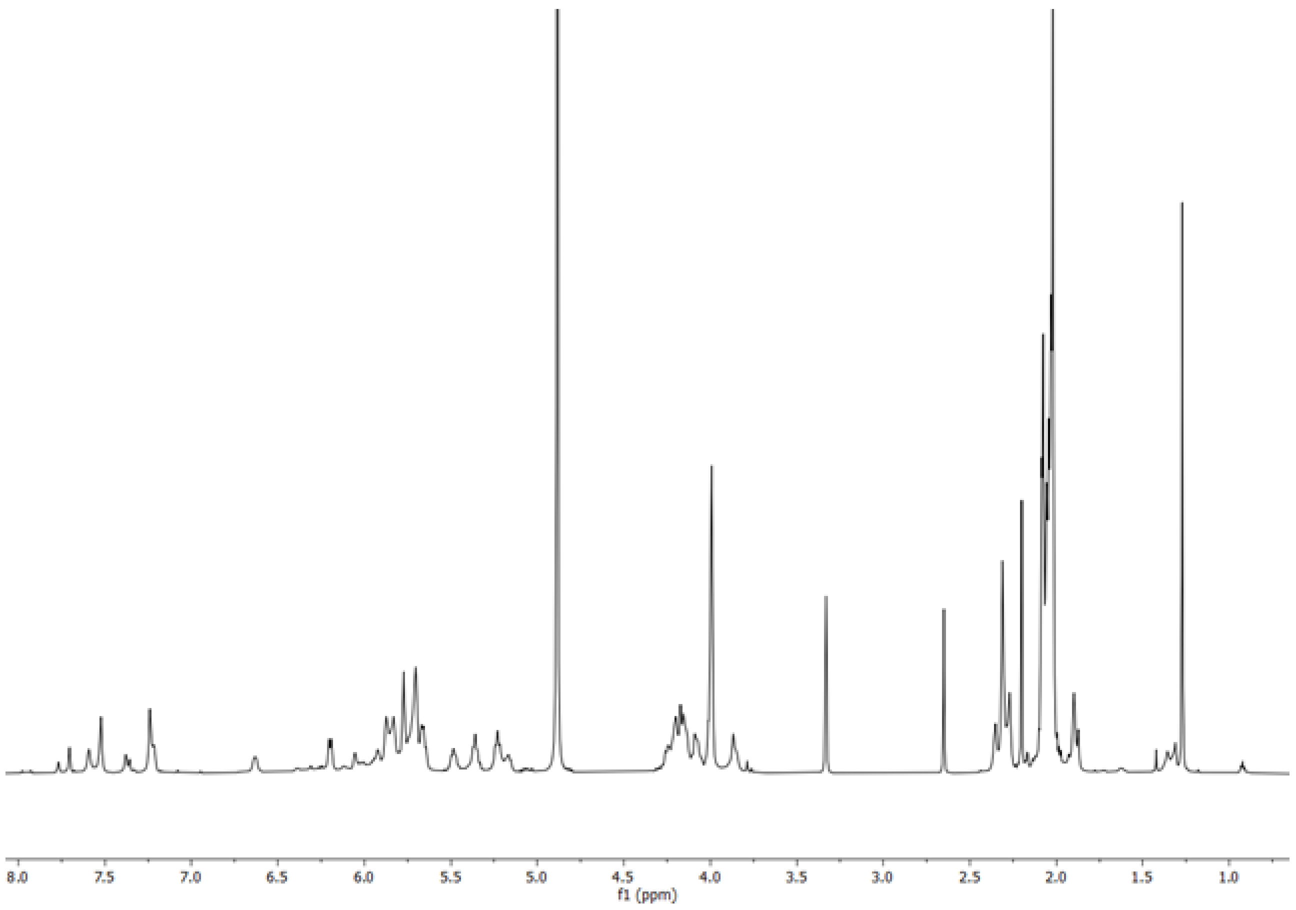

Figure S2. ^{1}H NMR spectrum of **Os-Tolu** in CD_3OD at 298 K

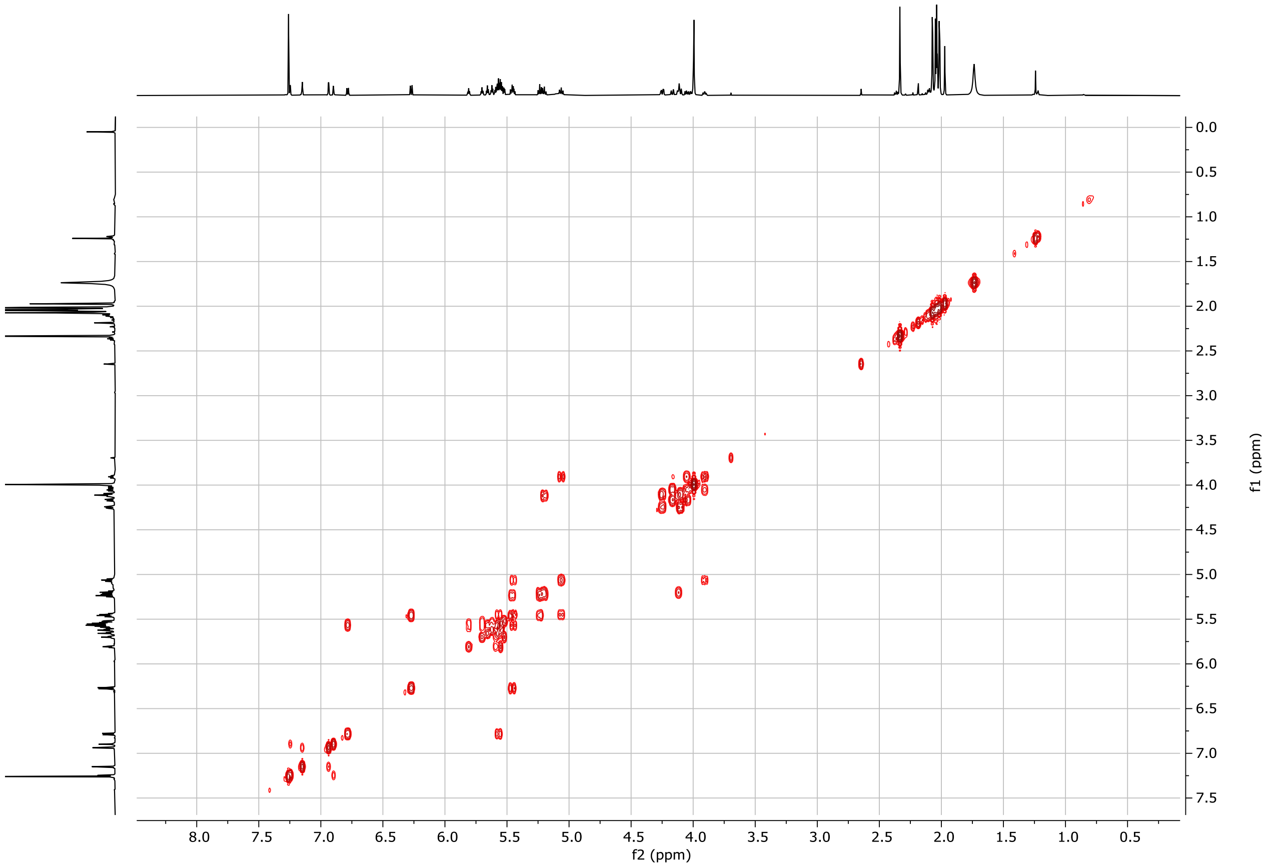

Figure S3. ^{1}H -^{1}H COSY NMR spectrum of **Os-Tolu** in $CDCl_3$ at 263 K

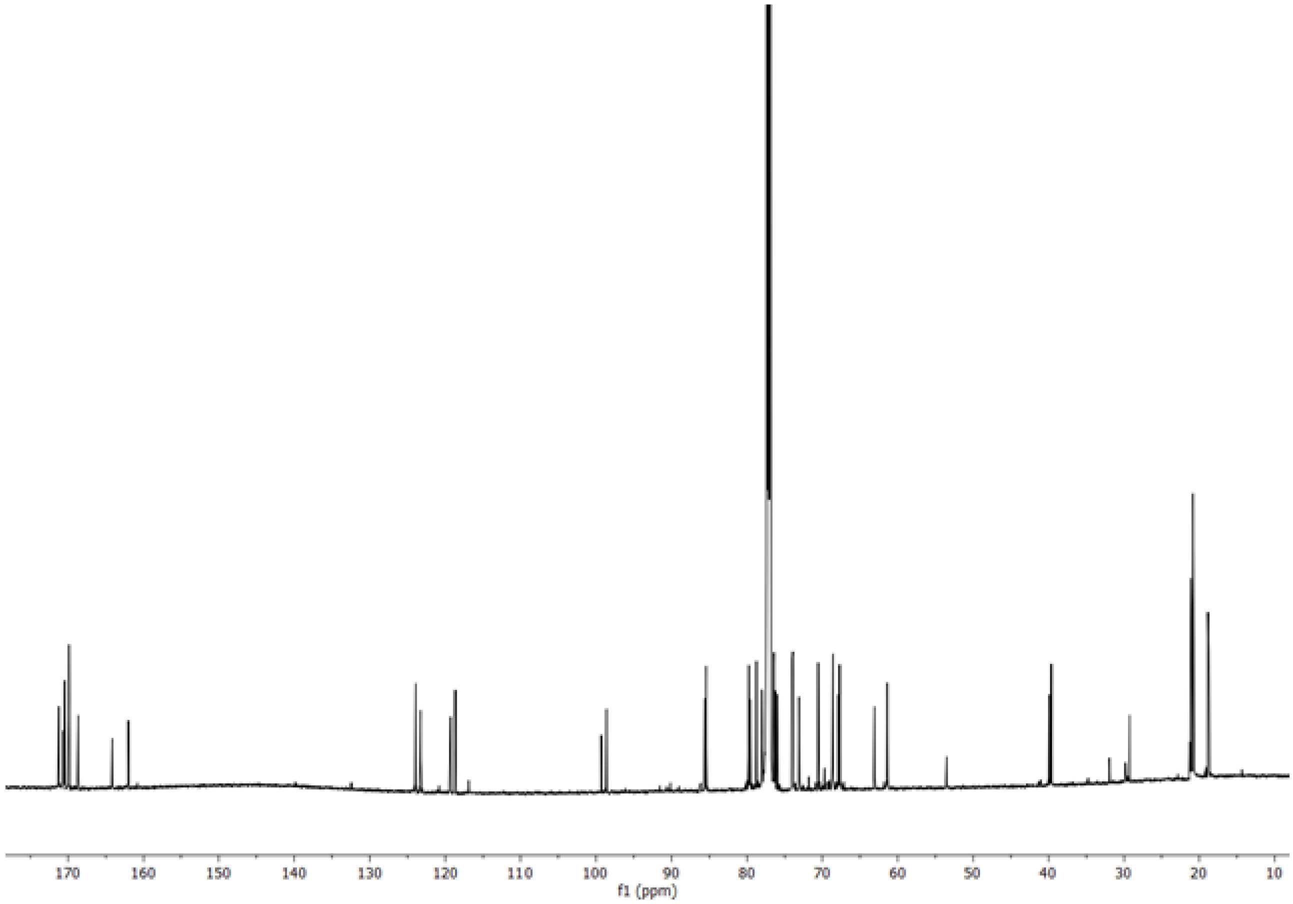

Figure S4. ^{13}C NMR spectrum of **Os-Tolu** in $CDCl_3$ at 263 K

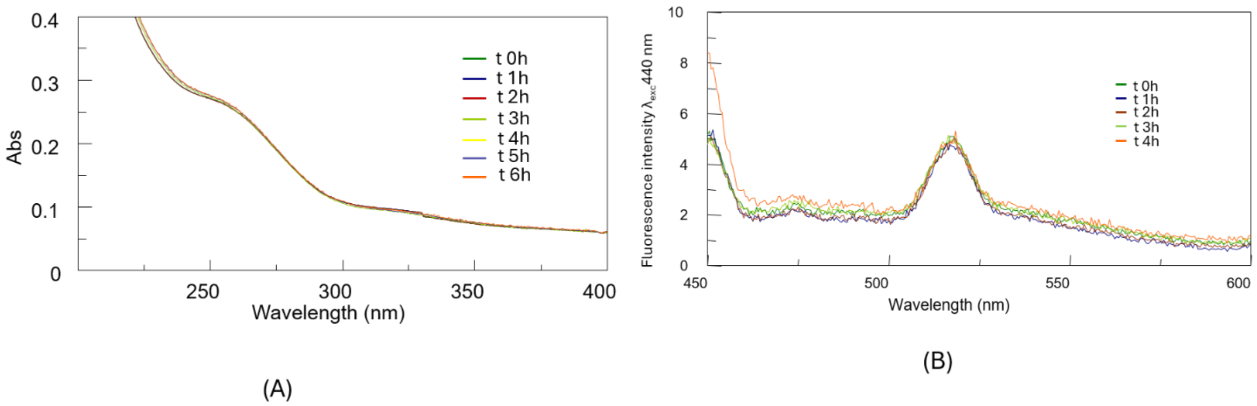

Figure S5. Overlay of (A) UV-vis spectra (B) fluorescence emission spectra: λ_{exc}= 440 nm of **Os-Tolu**.

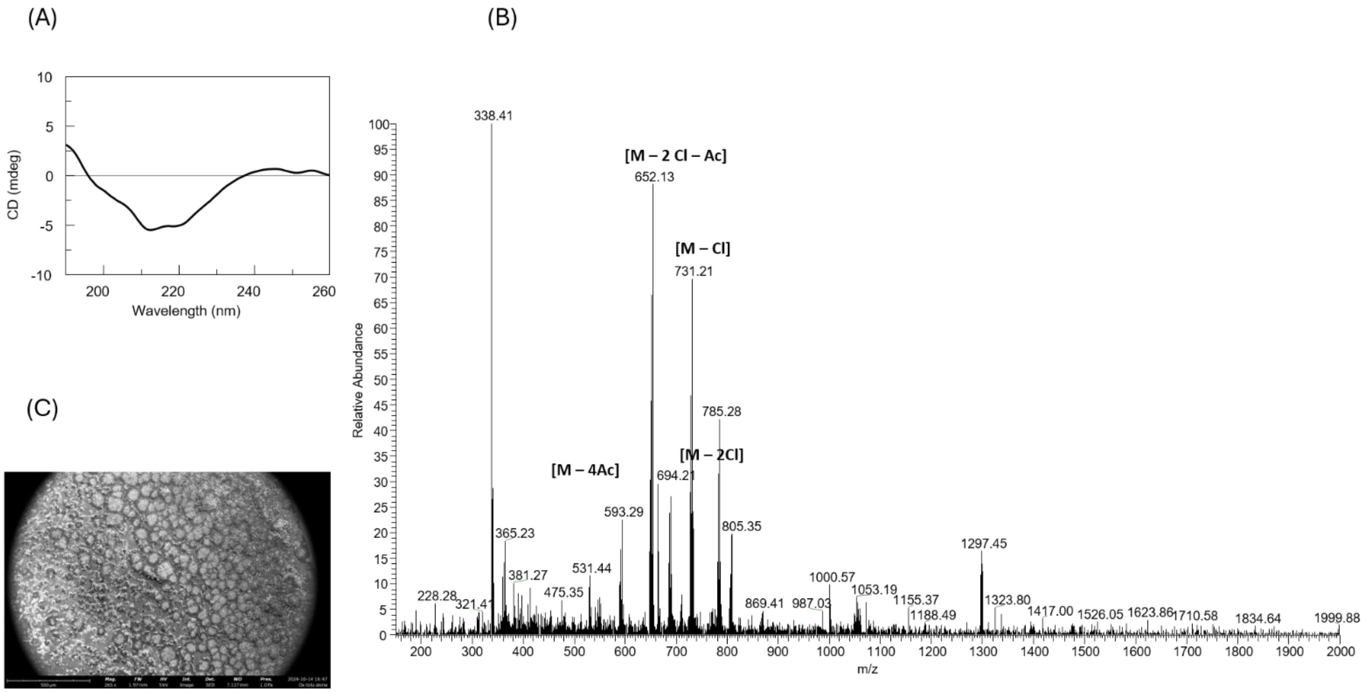

Figure S6. Os-Tolu (A) CD spectrum, (B) ESI-MS spectrum after t=2h of stirring and (C) SEM image at t=4h of stirring.

Table S1: Assigned adducts and related chemical formulas

**Assigned Adducts**	**Chemical Formula Adduct (MW amu)**
$NPM1_{264-277}$ + Os-Tolu -1Cl	$C_{107}H_{157}ClN_{24}O_{29}OsS$ (MW: 2501.32)
$NPM1_{264-277}$ + Os-Tolu -2Cl	$C_{107}H_{157}N_{24}O_{29}OsS$ (MW: 2465.86)
$NPM1_{264-277}$ + Os-Tolu -2Cl -1 Ac	$C_{105}H_{154}N_{24}O_{28}OsS$ (MW: 2422.82)

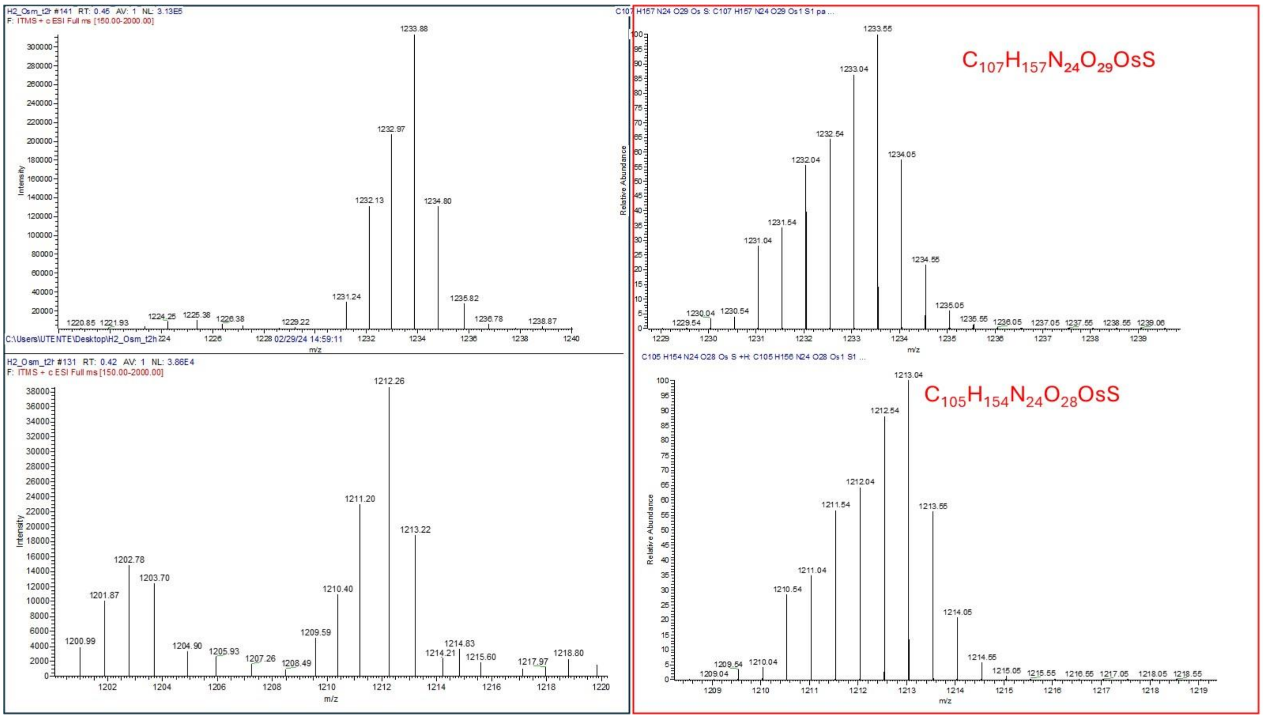

Figure S7: Comparison of m/z (z=+2) isotopic pattern of experimental (left black panel) and simulated (right red panel) spectra.
